# Prevalence, predictors and types of unpleasant and adverse effects of meditation in regular meditators: international cross-sectional study

**DOI:** 10.1192/bjo.2021.1066

**Published:** 2021-12-10

**Authors:** Luca Pauly, Niklas Bergmann, Inge Hahne, Sarah Pux, Eric Hahn, Thi Minh Tam Ta, Michael Rapp, Kerem Böge

**Affiliations:** Department of Psychiatry and Psychotherapy, Campus Benjamin Franklin, Charité – Universitätsmedizin Berlin, Germany; and Department for Clinical Psychology and Psychotherapy, Psychologische Hochschule Berlin, Germany; Department of Psychiatry and Psychotherapy, Campus Benjamin Franklin, Charité – Universitätsmedizin Berlin, Germany; Department of Psychiatry and Psychotherapy, Campus Benjamin Franklin, Charité – Universitätsmedizin Berlin, Germany; Department of Psychiatry and Psychotherapy, Campus Benjamin Franklin, Charité – Universitätsmedizin Berlin, Germany; Department of Psychiatry and Psychotherapy, Campus Benjamin Franklin, Charité – Universitätsmedizin Berlin, Germany; Department of Psychiatry and Psychotherapy, Campus Benjamin Franklin, Charité – Universitätsmedizin Berlin, Germany; Department for Social and Preventive Medicine, University of Potsdam, Germany; Department of Psychiatry and Psychotherapy, Campus Benjamin Franklin, Charité – Universitätsmedizin Berlin, Germany

**Keywords:** Mindfulness, meditation, third wave of cognitive–behavioural therapy, unpleasant events, adverse events

## Abstract

**Background:**

Meditation is commonly implemented in psychological therapies since the ‘third wave’ of cognitive–behavioural therapy has increased the focus on mindfulness-based interventions. Although extensive research literature demonstrates its benefits, little is known about potential adverse effects.

**Aims:**

The aim of this study is to report the prevalence, type and severity of particularly unpleasant meditation-related experiences in the largest cross-sectional study on this topic to date, with 1370 regular meditators.

**Method:**

The participants were asked whether they ever encountered particularly unpleasant experiences as a result of their meditation experience. For the first time, the type and severity of those experiences were assessed and the association with several predictors, such as pre-existing mental disorders, were explored via logistic and linear regression.

**Results:**

Similar to previous studies, 22% of participants (95% CI 20–24) reported having encountered unpleasant meditation-related experiences, and 13% of participants (95% CI 3–5) reported experiences that were categorised as adverse. Those were mostly of affective, somatic and cognitive nature. Unpleasant meditation-related experiences were more likely to occur in participants with pre-existing mental illnesses (*P* = 0.000, 95% CI 1.25–2.12).

**Conclusions:**

This study demonstrates that unpleasant meditation-related experiences are prevalent among meditators and, to a relevant extent, severe enough to warrant further scientific inquiry. Longitudinal studies are needed to examine whether the unpleasant meditation-related experiences are merely negative and thus should be avoided, or are an inherent part of the contemplative path.

To date, research has primarily focused on the benefits of meditation on health and well-being, such as a reduction of anxiety,^1^ stress^2^ and depressive symptoms.^3^ Less than a quarter of studies on meditation assessed possible adverse effects, which leads to a probable underestimation.^4,5^ A recent larger-scale, cross-sectional online study by Schlosser et al, on unpleasant meditation-related experiences, indicated that 25.6% of regular meditators reported having had particular unpleasant meditation-related experiences.^6^ Nevertheless, the authors did not assess the type and severity of those experiences. Other recent studies found that between 50 and 53% of meditators reported at least one meditation-related adverse effect,^7,8^ and 6–14% reported enduring negative adverse effects.^7^ However, as shown by a large systematic review, there is a high level of heterogeneity in the existing studies, regarding the prevalence of meditation-related adverse effects.^9^ Given the rising popularity of meditation practices in self-help and therapeutic settings, prevention of harm is a primary ethical duty that requires comprehensive knowledge of possible adverse effects.

## Defining unpleasant and adverse meditation-related effects

It remains largely unclear how to define particularly unpleasant meditation-related experiences. This is partially because of the arbitrary distinction between uncomfortable experiences that are yet part of the contemplative process, and merely harmful experiences with lasting consequences. Regarding the great heterogeneity of terms for unpleasant effects used in pre-studies,^9^ there is a need for more conceptual clarity to facilitate more systematic research. Duggan et al^10^ define harm in psychological treatments as ‘a sustained deterioration that is caused directly by the psychological intervention’. In contrast, the World Health Organization defines harm on a continuum that includes suffering or impairment of function of any duration, including experiences that are ‘subjectively unpleasant’.^11^ Linden^12^ proposed a taxonomy for unpleasant effects of psychological treatment, with ‘unwanted effects’ being all negative events that occur in the wake of a treatment. Side-effects are defined as adverse reactions that are caused by correct, not maladaptive, treatment. The author proposed a five-point Likert scale to assess the severity of those effects. In the present study, this scale served as a tool to distinguish between temporary unpleasant and adverse effects. The umbrella term ‘unpleasant meditation-related experiences’ was chosen over the label ‘unwanted effects’, which excludes unpleasant but wanted effects, as they are essential for the wider benefits of meditation practices to unfold. Because of the cross-sectional design of the study, it cannot be assessed whether the meditation practice was applied correctly. Therefore, the term side-effect, as defined by Linden,^12^ was not adopted.

## Aim of the present study

The aim of this study is to report the prevalence, type and severity of particularly unpleasant meditation-related experiences in a large international sample. The study aims to replicate the results of Schlosser et al^6^ and, for the first time, explore the association of unpleasant meditation-related experiences with neuroticism and pre-existing mental illness. The purpose of this study is to ensure better education and application of mindfulness interventions and prevent potential harm.

## Hypotheses

Based on the study by Schlosser et al,^6^ it is assumed that meditation types that belong to the category ‘deconstructive meditation’ are more likely to be associated with unpleasant meditation-related experiences than non-deconstructive meditation types. In their study, they could also show that experience of a meditation retreat is associated with unpleasant meditation-related experiences. Those effects shall be replicated here and further extended by defining the severity of those experiences. Our first hypothesis is that people who practice only deconstructive meditation-types report unpleasant meditation-related experiences in higher frequency and in higher severity than people who practice only non-deconstructive meditation types. Our second hypothesis is that people who have attended a meditation retreat report unpleasant meditation-related experiences in higher frequency and severity than people who have never attended a meditation retreat.

Mindfulness is defined as the capacity to see mental events as transient, to let difficult cognitions pass without ruminating about them and to accept difficult thoughts without judging them.^1^ It is assumed that a higher degree of mindfulness is serving as a buffer against unpleasant meditation-related experiences. Therefore, our third hypothesis is that higher levels of mindfulness (measured with the Mindfulness Attention Awareness Scale; MAAS) are negatively associated with the occurrence and severity of unpleasant meditation-related experiences.

It has been shown that repetitive negative thinking is associated with symptoms of depression and anxiety.^13^ Schlosser et al^6,14^ indicated that repetitive thinking is positively associated with the occurrence of unpleasant meditation-related experiences. Based on these results, it is expected that higher degrees of repetitive negative thinking facilitate the occurrence of meditation-related negative experiences and are associated with higher severity of those experiences. Our fourth hypothesis is that higher levels of repetitive negative thinking (measured with the Perseverative Thinking Questionnaire; PTQ) are positively associated with the occurrence and severity of unpleasant meditation-related experiences.

Positive correlations of neuroticism with negative affect, anxiety and stress, as well as negative correlations with well-being and mindfulness, have been found.^15–17^ A higher degree of neuroticism is expected to favour the occurrence of meditation-induced negative experiences. It is further hypothesised that a higher degree of neuroticism is associated with higher severity of those experiences, as neurotic people are more likely to respond to unpleasant experiences to aggravate the severity of adverse outcomes.^18^ Therefore, our fifth hypothesis is that high levels of neuroticism (measured with the Big Five Inventory; BFI) are positively associated with the occurrence and severity of unpleasant meditation-related experiences.

Finally, and for the first time, the role of pre-existing mental disorders will be explored in relation to the occurrence and severity of unpleasant meditation-related experiences.

## Method

### Design and procedure

The study used a cross-sectional design. An online survey was developed on the platform Unipark for MacOS (Tivian XI GmbH, Cologne, Germany; see https://www.unipark.com/). The link to the questionnaire was sent to international meditation and Buddhist centres, meditation teachers, therapists that teach Mindfulness-based Stress Reduction and other mindfulness communities. It was shared on Instagram and Facebook, and platforms to recruit participants, such as PollPool and SurveyCircle. The participants were informed about the study's aims to provide novel insights into possible influences of meditation practices and their relationship to cognitive and emotional processes within the online questionnaire. Written informed consent to the collection and processing of their data was obtained from all respondents. All procedures contributing to this work comply with the ethical standards of the relevant national and institutional committees on human experimentation and with the Helsinki Declaration of 1975, as revised in 2008. All procedures involving humans were approved by the ethics committee of the Charité – Universitätsmedizin Berlin, Campus Benjamin Franklin (approval number ES4/127/20). This study was preregistered at https://aspredicted.org/pm34k.pdf (no. 42195).

### Participants

Participants were recruited between June 2020 and May 2021 to participate in the anonymous online survey that took approximately 20–25 min to complete. The inclusion criteria were consent to the data collection and processing, good understanding of the English language and a minimum age of 18 years. There were no exclusion criteria. A total of 11 143 individuals started the survey, of which 1760 completed it. In the next step, a total of 390 participants were excluded from the analyses: 29 indicated an age <18 years, 148 had no meditation experience, 52 indicated only yoga practices, 11 did not demonstrate meditation practices, 140 had <2 months of meditation experience and 10 practised less than once a week. Therefore, 1370 regular meditators were included in the subsequent analyses. Initially, demographic information, such as age, gender, place of residence and belief, were assessed. Participant characteristics are displayed in Table 1.

**Table 1:** Demographic and meditation-related characteristics of 866 regular meditators

	Missing values, *n* (%)	Summary statistic, *n* (%)^a^
Age, years, mean (s.d.)	0	42.36 (15.6)
Gender	1 (0.1%)	
Female		907 (66.2%)
Belief	1 (0.1%)	
Religious		527 (38.4%)
Other		323 (23.6%)
Agnostic		71 (5.2%)
Atheist		123 (9.0%)
Nothing in particular		325 (23.7%)
Continent of residence	2 (0.1%)	
Europe		831 (60.7%)
Asia		143 (10.4%)
North America		275 (20.1%)
Australia and New Zealand		76 (5.5%)
South America		27 (2.0%)
Africa		16 (1.2%)
Place of domicile	4 (0.3%)	
Megacity		93 (6.8%)
Large metropolitan area (1.5–10 million people)		337 (24.6%)
Metropolitan area (500 000–1.5 million people)		236 (17.2%)
Medium urban area (100 000–500 000 people)		305 (22.3%)
Small urban area (10 000–100 000 people)		232 (16.9%)
Rural area (<10 000 people)		163 (11.9%)
Meditation practice variables		
Lifetime meditation experiences, years, median (interquartile range)	0	5 (1.0–13.3)
Meditation intensity, hours per week, mean (s.d.)	0	6.9 (13.4)
Retreat experience (at any point in life)	0	787 (57.4%)
Meditation context^b^	5 (0.4%)	
Guided by teacher		342 (25%)
Guided by app, video or voice via the internet		594 (43.4%)
Self-instructed without any guidance		1009 (73.7%)
Group setting without a teacher		227 (16.6%)
Meditation types^b^	283 (20.7%)	
Attentional		1033 (75.5%)
Constructive		647 (47.3%)
Deconstructive		636 (46.5%)
Pre-existing mental disorder before meditating	0	444 (32.4%)
Particularly unpleasant meditation-related experiences	0	301 (22.0%)
Type of unpleasant meditation-related experience^b^	1068 (78%)	
Cognitive unpleasant experience		158 (11.5%)
Perceptual unpleasant experience		107 (7.8%)
Affective unpleasant experience		223 (16.3%)
Somatic unpleasant experience		183 (13.4%)
Conative unpleasant experience		89 (6.5%)
Unpleasant experience regarding sense of self		136 (9.9%)
Social unpleasant experience		98 (7.2%)
Severity of unpleasant experience, mean (s.d.)	1070 (79%)	1.84 (0.9)
Mild, without consequences		122 (8.9%)
Moderate, distressing		120 (8.8%)
Severe, in need of countermeasures		43 (3.1%)
Very severe, lasting consequences		12 (0.9%)
Extremely severe, life-threatening consequences or requiring hospital admission		3 (0.2%)
Mindfulness, mean (s.d.)	0	4.3 (0.9)
Repetitive negative thinking, mean (s.d.)	0	2.5 (0.7)
Neuroticism, mean (s.d.)	0	2.5 (0.9)

a. Statistics in this column are *n* (%) unless otherwise specified.

b. Participants could choose more than one option; the total percentage might exceed 100%.

### Meditation experience

The participants were asked to report their length of meditation experience in years, meditation frequency in hours per week, their usual meditation context and if they ever attended a retreat. Then, the participants determined the type of meditation they practised by choosing the ones that applied from a given set of options. The meditation types provided were based on the taxonomy by Dahl et al,^19^ categorising them into attentional, constructive and deconstructive types. Attentional meditation types mainly focus attention on phenomena such as the breath. Constructive practices, such as loving-kindness meditation, primarily aim to consolidate emotional patterns such as empathy. Deconstructive meditation types, such as *Vipassana*, aim to dissolve the implicit belief in the inherent and independent existence of objects of consciousness, including views of the self and others.

### Pre-existing mental disorders

We assessed whether participants had a history of pre-existing mental disorders. To clarify the time of disease onset, participants were directly asked if they ever had a mental disorder before they started to practice meditation.

### Meditation-related unpleasant and adverse effects

To assess adverse effects, the same question was applied as in the study by Schlosser et al^6^: ‘Have you ever had any particularly unpleasant experiences which you think may have been caused by your meditation practice?’. As an extension of the study, the severity of the unpleasant experience was assessed by the five-point Likert scale from Linden,^12^ whereby 1 is defined as a mild effect without consequences; 2 is a moderate, distressing effect; 3 is a severe effect in need of countermeasures; 4 is a very severe effect with lasting consequences and 5 is an extremely severe effect with life-threatening consequences or requiring hospital admission. This scale served as the classification for unpleasant experiences and adverse effects. Mild effects (1 on the Likert scale) should be understood and avoided when possible, but do not appear to meet definitions of harm.^10^ They are referred to as ‘unpleasant effects’ in the current study. In contrast, moderate, severe, very severe and extremely severe (2–5 on the Likert scale, respectively) effects were categorised as ‘adverse effects’, as they indicate some kind of suffering or impairment in function.^11^ Furthermore, the exact type of unpleasant experience was assessed. The seven categories of meditation-related adverse effects that previously had been identified were applied: cognitive, perceptual, affective, somatic, conative sense of self and social.^20^ The participants could choose every category if the experience was of that nature.

### Mindfulness

Mindfulness was measured with the validated MAAS.^21^ The MAAS is a 15-item measure that uses a six-point Likert scale. The scale is unidimensional and measures the capacity to be mindful in daily life, mindful awareness of distressing thoughts and images, and the capacity to observe mental events as transient. The MAAS has displayed good psychometric properties, with a Cronbach's alpha of 0.89–0.93.^22^

### Repetitive negative thinking

Repetitive negative thinking was measured with the PTQ,^13^ which is a 15-item measure that uses a five-point Likert scale. Participants were asked to indicate how they typically think about negative experiences or problems, with higher scores indicating higher levels of repetitive negative thinking. The PTQ has displayed excellent psychometric properties across samples: Cronbach's alpha ranged from 0.94 to 0.95.^13^

### Neuroticism

Neuroticism was measured with the BFI,^23^ entailing 44 items that assess how participants see themselves, using a five-point Likert scale. The BFI items displayed high internal reliability across samples, with a Cronbach's alpha of 0.86 for the neuroticism factor.^24^

### Statistical analysis

Subsequent analyses were performed with IBM SPSS Statistics version 24 for MacOS. First, Pearson correlation coefficients were calculated to test whether the variables are associated. Logistic and linear regression models were fitted to assess the association between occurrence and severity of particularly unpleasant meditation-related experiences and age (continuous), gender (binary: female/male), belief (binary: belief/no belief), pre-existing mental disorders (binary: yes/no), lifetime meditation experience (continuous) and meditation intensity (continuous). To harmonise our approach with Schlosser et al,^6^ a binary variable to denote deconstructive (*n* = 30) and non-deconstructive (*n* = 451) meditation types was generated (meditation type). In contrast to Schlosser et al, we did not compare religious and non-religious participants, as a high percentage of participants stated ‘other’ as their belief, mainly indicating some spiritual belief. Thus, a binary variable to denote participants with any kind of belief and those with no belief was generated (belief versus no belief). The belief group (*n* = 850) included participants that indicated a religion or ‘other’ as a belief, whereas the no-belief group (*n* = 519) included those that answered as agnostic, atheist or ‘nothing in particular’.

To test the hypotheses, binary logistic and linear regression models were applied. The logistic regression models included the occurrence of unpleasant meditation-related experiences (yes/no) as a binary outcome variable. The linear regression models include the severity of these experiences as a metric outcome variable. The predictors that were included in the regression models were the meditation type (binary: deconstructive/non-deconstructive), experience of a meditation retreat (binary: yes/no), mindfulness (continuous), repetitive thinking (continuous) and neuroticism (continuous).

## Results

To test the hypotheses, a sample of at least *N* = 717 people who completed the study was required to reach sufficient power of 0.80. The power calculation was carried out using the program ‘g-power’. Participant characteristics and descriptive statistics are displayed in Table 1.

In total, 22% of all participants reported having unpleasant meditation-related experiences. Regarding the severity, 8.9% of all participants (*n* = 122) indicated mild experiences without consequences, which were categorised as unpleasant but temporary meditation-related effects. Further, 8.8% (*n* = 120) reported moderate adverse effects, 3.1% (*n* = 43) reported severe effects in need of countermeasures, 0.9% (*n* = 12) reported very severe effects with lasting consequences and 0.2% (*n* = 3) reported extremely severe effects with life-threatening consequences or requiring hospital admission. Moderate to extremely severe experiences were categorised as adverse effects, which were reported by 13% in total.

Table 2 displays all logistic regression models. Strong evidence was found that people with a mental disorder before they started meditating had higher odds of having particularly unpleasant meditation-related experiences (odds ratio 1.63, 95% CI 1.25–2.12, *P* = 0.000). To control for possible confounding, pre-existing mental disorders were included as covariates in the subsequent analysis.

**Table 2:** Associations with the occurrence of unpleasant meditation-related experiences

Predictors	% (*n*)^a^	*β* coefficient	Odds ratio^b^	95% CI	*P*-value
Age	−	−0.00	0.99	0.99–1.01	0.609
Gender					
Male	21.4% (96)				
Female (versus male)	22.3% (202)	0.047	1.05	0.80–1.38	0.740
Belief					
No belief	8.6% (118)				
Belief (versus no belief)	13.4% (183)	0.07	1.07	0.83–1.39	0.601
Pre-existing mental disorders					
No pre-existing mental disorders	19.1% (177)				
Pre-existing mental disorders (versus no pre-existing mental disorders)	27.9% (124)	0.49	1.63	1.25–2.12	0.000**
Lifetime meditation experience, years	−	0.00	1.00	0.99–1.00	0.758
Meditation intensity, hours per week	−	−0.005	1.00	0.98–1.01	0.370
Meditation type					
Non-deconstructive	15.7% (71)				
Deconstructive only (versus non- deconstructive)	23.3% (7)	0.49	1.63	0.67–3.94	0.279
Retreat experience (at any point in life)					
No experience	16.2% (94)				
Experience (versus no experience)	26.3% (207)	0.63	1.89	1.43–2.48	0.000*
Mindfulness	−	−0.07	0.94	0.81–1.08	0.367
Repetitive negative thinking	−	0.29	1.34	1.12–1.60	0.002**
Neuroticism	−	0.26	1.29	1.13–1.48	0.000**

a. No summary statistics are presented for continuous predictors.

b. For binary explanatory variables (gender, belief, meditation type, retreat experience), the estimate describes the odds of occurrence of particular unpleasant meditation-related experiences in one group relative to the reference category (indicated in parentheses). For continuous explanatory variables (age, meditation experience, meditation intensity, mindfulness, repetitive negative thinking, neuroticism), the estimate reflects the expected increase in the odds of particularly unpleasant meditation-related experiences per one-unit increase in the explanatory variable.

* *P* < 0.05, **P < 0.01.

Table 3 displays all linear regression models. People with pre-existing mental disorders also indicated significantly higher severity of unpleasant meditation-related experiences (*ß* = 0.38, s.e. = 0.10, 95% CI 0.18–0.58, *P* = 0.000).

**Table 3:** Associations with the severity of unpleasant meditation-related experiences

Predictors	*ß*	s.e.(*ß*)	95% CI	*R*²	*P*-value
Age	−0.01	0.00	−0.01 to 0.00	0.01	0.076
Gender^a^	−0.04	0.10	−0.25 to 0.16	0.00	0.671
Belief^b^	0.18	0.10	−0.02 to 0.39	0.01	0.079
Pre-existing mental disorders^c^	0.38	0.10	0.18–0.58	0.05	0.000*
Lifetime meditation experience	0.00	0.00	0.00–0.00	0.00	0.345
Meditation intensity, hours per week	0.01	0.01	0.00–0.02	0.01	0.062
Meditation type^d^	−0.19	0.10	−0.39 to 0.01	0.01	0.060
Retreat experience^e^ (at any point in life)	−0.09	0.11	−0.30 to 0.13	0.00	0.447
Mindfulness	−0.12	0.06	−0.23 to –0.01	0.01	0.041*
Repetitive negative thinking	0.20	0.08	0.49–0.34	0.02	0.009**
Neuroticism	0.13	0.06	0.02–0.24	0.02	0.022*

a. Gender is encoded as 0 = female, 1 = male.

b. Belief is encoded as 0 = no belief, 1 = belief.

c. Pre-existing mental disorders are encoded as 0 = no pre-existing disorders, 1 = pre-existing disorders.

d. Meditation type is encoded as 0 = only non-deconstructive types, 1 = only deconstructive types.

e. Retreat experience is encoded as 0 = no retreat experience, 1 = retreat experience.

* *P*< 0.05, **P < 0.01.

No evidence was found for significantly higher odds of people practicing only deconstructive meditation types (odds ratio 1.63, 95% CI 0.67–3.94, *P* = 0.279). The meditation type was not a significant predictor for the severity of unpleasant meditation-related experiences (*ß* = −0.19, s.e. = 0.10, 95% CI −0.39 to 0.01, *P* = 0.060).

Significant evidence was found that the odds of unpleasant meditation-related experiences were 88.5% higher in meditators who had attended a retreat compared with meditators who never had attended a retreat (odds ratio 1.89, 95% CI 1.43–2.48, *P* = 0.000). This association was even consolidated after adjusting for pre-existing mental disorders (odds ratio 1.94, 95% CI 1.49–2.60, *P* = 0.000). No evidence was found for an association between experience of a retreat and severity of unpleasant meditation-related effects (*ß* = −0.09, s.e. 0.11, 95% CI −0.30 to 0.13, *P* = 0.447).

The association between mindfulness and occurrence of particularly unpleasant meditation-related experiences was not significant (odds ratio 0.94, 95% CI 0.81–1.08, *P* = 0.367). Evidence was found for a negative association between mindfulness and the severity of those unpleasant experiences (*ß* = −0.12, s.e. = 0.06, 95% CI −0.23 to −0.01, *P* = 0.041). After including pre-existing mental illnesses as a covariate, the association was no longer significant (*ß* = −0.07, s.e. = 0.06, 95% CI −0.18 to 0.05, *P* = 0.255).

Significant evidence was found that for every unit increase in repetitive negative thinking, the odds of reporting unpleasant experiences increased by 33.6% (odds ratio 1.34, 95% CI 1.12–1.60, *P* = 0.002). The association was slightly attenuated after adjusting for pre-existing mental disorders (odds ratio 1.24, 95% CI 1.03–1.49, *P* = 0.024). The association between repetitive negative thinking and the severity of unpleasant meditation-related experiences was also significant (*ß* = 0.20, s.e. = 0.08, 95% CI 0.49–0.34, *P* = 0.009). However, this association was no longer significant after adjusting for pre-existing mental disorders (*ß* = 0.13, s.e. = 0.08, 95% CI −0.03 to 0.28, *P* = 0.102).

Strong evidence was found that for every unit increase in neuroticism, the odds of unpleasant meditation-related experiences increased by 29.3% (odds ratio 1.29, 95% CI 1.13–1.48, *P* = 0.000). This association was only slightly attenuated after adjusting for pre-existing mental disorders (odds ratio 1.21, 95% CI 1.05–1.40, *P* = 0.010). Neuroticism was also associated with the severity of unpleasant meditation-related experiences (*ß* = 0.13, s.e. = 0.06, 95% CI 0.02–0.24, *P* = 0.022). The association was no longer significant after adjusting for pre-existing mental disorders (*ß* = 0.05, s.e. = 0.06, 95% CI −0.07 to 0.17, *P* = 0.403).

## Discussion

The current study is the largest cross-sectional study to date that examines unpleasant meditation-related experiences and influential factors such as pre-existing mental disorders and neuroticism. Overall, 22% (*n* = 301) of the participants indicated previously having encountered unpleasant meditation-related experiences. Those numbers are in line with rates found in previous studies that used a similar open-ended question format,^6,25–28^ whereas studies using systematic monitoring of unpleasant experiences found higher rates.^7,8^ Regarding the severity, the results are consistent with those from a recent large population-based survey,^8^ which showed that 7.1% of participants reported some functional impairment, 2.3% had moderate impairment and 0.2% had severe functional impairment from their meditation practice. In line with previous research,^9,20^ the reported unpleasant effects were mostly of affective, somatic and cognitive nature. No analyses were conducted regarding a more specific analysis of the seven different types of adverse effects. Future research could specifically address this. The study indicates that unpleasant meditation-related experiences are prevalent enough among meditators to warrant further scientific inquiry.

### Pre-existing mental illness

Importantly, it could be shown that participants with pre-existing mental disorders were more likely to report unpleasant meditation-related experiences as well as a higher severity of those experiences. As the onset of the mental illness had been before they started to meditate, it can be assumed that the mental disorders preceded the unpleasant experiences and not the other way around. In previous studies, pre-existing mental disorders were often not assessed; thus, this represents an important finding. People with mental disorders could tend to overidentify with unpleasant emotions, being unable to let go of them, and thus enhancing the severity and resulting distress of unpleasant meditation-related experiences. Participants with pre-existing mental disorders could also seek to meditate to manage their symptoms better and consolidate a more productive way of handling unpleasant emotions. Practising meditation may cause vulnerable individuals to be more aware of their sensations, emotions and thoughts,^29^ resulting in the increased reporting of unpleasant experiences seen in the current study. Because of the correlational nature of this study, we cannot draw definite conclusions about the causality of this finding. However, it is crucial to point out that in the current study, people with pre-existing mental illnesses were more likely to report meditation-related adverse effects. This is even after accounting for the negative correlation between mindfulness and severity of adverse meditation-related effects, which was no longer significant after controlling for pre-existing mental illnesses. Accordingly, being more mindful does not seem to serve as a protective factor against meditation-related adverse effects for people with pre-existing mental illnesses. This finding highlights pre-existing mental disorders as a possible risk factor for adverse meditation-related effects.

### Meditation type

Contrary to Schlosser et al,^6^ no evidence was found concerning an association of unpleasant meditation-related experiences and deconstructive meditation types. It is crucial to mention that the percentage of participants only practising deconstructive meditation types was relatively small (2.2%, *n* = 30), as the majority indicated deconstructive as well as non-deconstructive meditation types (64.9%, *n* = 889). To conclude the role of meditation type, a larger sample of only deconstructive practice types is required in future.

### Experience of a meditation retreat

As hypothesised, meditators who had attended a meditation retreat were more likely to report unpleasant experiences. Retreat-specific characteristics presumably represent a context that makes unpleasant experiences more likely. This involves long and frequent sessions, silence, limited access to distractions, a strict schedule with a limited amount of sleep and often a change in diet (e.g. vegan). Another explanation could be that meditators who encounter unpleasant experiences participate in retreats to receive further guidance and supervision in their meditation practice. As there was no evidence found for higher severity of unpleasant experiences in people who had attended a retreat, definite conclusions about the effects of retreats cannot be drawn.

### Repetitive negative thinking

Strong evidence was found for a positive association between repetitive negative thinking and the occurrence and severity of unpleasant experiences. Participants with heightened levels of repetitive negative thinking possibly find it harder to disengage from unpleasant thoughts that can arise during meditation. They tend to respond with rumination,^13^ leading to increased distress. However, after controlling for pre-existing mental illnesses, the association between repetitive negative thinking and the severity of unpleasant meditation-related experiences was no longer significant. Preservative negative thinking is a transdiagnostic process that manifests across a wide spectrum of mental health disorders, including depression, obsessive–compulsive disorder, panic disorder and psychosis.^13,30^ Therefore, it could be assumed that pre-existing mental illness determines the association of repetitive negative thinking and unpleasant meditation-related experiences.

### Neuroticism

Neuroticism was found to be a strong predictor for occurrence and severity of unpleasant meditation-related experiences. This is the first time the role of neuroticism has been examined regarding unpleasant meditation-related experiences. The personality trait of neuroticism refers to relatively stable tendencies to respond with negative emotions to threat or frustration.^16^ Indeed, many studies examining the relationship between negative affectivity and adverse outcomes focus on traits that might be considered facets of neuroticism, such as trait hostility and anger.^18^ The most evident explanation for the strong association found is that people with high levels of neuroticism are more likely to respond anxiously to negative thoughts that can arise in meditation. However, when controlled for pre-existing mental illnesses, neuroticism was no longer a significant predictor for the severity of unpleasant meditation-related experiences. Many pre-existing mental disorders are strongly associated with neuroticism,^16,31,32^ and seem to play a mediating role regarding the association of neuroticism and severity of unpleasant meditation-related experiences.

### Limitations

Because of the cross-sectional nature of the study, no conclusions about causality can be drawn. The exact time and context in which the unpleasant experiences occurred were not assessed; thus, they could have taken place a long time ago, whereas variables such as mindfulness and repetitive negative thinking captured the status quo. Further, occurrence, type and severity of unpleasant effects were each assessed by only one question, limiting the reliability of the data. Open-ended question formats seem to underestimate the prevalence of adverse effects by nearly 70%, which could have led to an underreporting of adverse effects.^7^ The questionnaire involved self-reports that were not previously validated. Participants self-attributed the unpleasant experiences to their meditation practise; however, this attribution is subjective, and it is not entirely clear whether the unpleasant experience might have also arisen without the meditation practice. Also, there may be a memory bias as most of the effects might have occurred some time ago, and may be forgotten and thus underreported. Because of the cross-sectional design, we do not know whether the meditation practice was applied correctly or maladaptively. We did not assess participant factors such as goals and expectations of meditation and general health behaviour, which were found to play a role in previous studies.^20^ Another limitation of our sample is that only 15.8% of participants who started the survey completed it. This presumably can be assigned to the rather long protocol and could have led to selection bias. Furthermore, the positive effects of meditation were not assessed; therefore, no conclusion can be drawn from the data about the relation of cost and benefits of meditation practices. If essential unpleasant meditation-related experiences are avoided, some of the benefits of the practice could potentially be removed; however, if non-essential unpleasant meditation-related experiences are somehow cultivated, this could potentially lead to unnecessary suffering.

### Conclusion and implications

This study is the first to examine those factors in such a large sample, and so the results should not be interpreted as conclusive, but should warrant further investigation. The data is suggestive of an interaction-based model where meditation practice on its own may lead to unpleasant experiences, but the specific type of effect and associated impairment is influenced by a number of factors. The findings suggest that there is no specific personality type that is prone to adverse effects of meditation, but that other factors such as mental illness are more decisive. Therefore, mindfulness-based interventions should be carefully adapted to the diagnosis and implemented by trained therapists to avoid possible adverse meditation-related experiences. This approach can be seen with dialectic behavioural therapy for borderline disorder,^33^ mindfulness-based cognitive therapy for depression,^34^ and mindfulness-based group therapy for psychosis.^35^ Regarding the rising popularity of meditation in a self-help context, those findings highlight the importance of safeguarding beyond clinical trials, potentially by making it mandatory to have an educational statement about possible adverse effects and risk factors of mediation in meditation apps. Long-term studies are needed to further investigate the nature of unpleasant and adverse meditation-related experiences and causal influences. Further, a standardised approach to define and assess the exact type, duration and severity of unpleasant meditation-related experiences is necessary. The current study serves as a guide for distinguishing and measuring unpleasant and adverse effects of meditation. Future studies should consider an interdisciplinary dialogue between Buddhist, scientific and clinical camps to advance the field and broaden the knowledge about possible harm and contraindications of meditation.

## Data Availability

The data that support the findings of this study are available from the corresponding author, K.B., upon reasonable request.

## References

[1] Hofmann S, Sawyer A, Witt A, Oh D. The effect of mindfulness-based therapy on anxiety and depression: a meta-analytic review. J Consult Clin Psychol 2010; 78(2): 169–83. 20350028 10.1037/a0018555 PMC2848393

[2] Khoury B, Lecomte T, Fortin G, Masse M, Therien P, Bouchard V, Mindfulness-based therapy: a comprehensive meta-analysis. Clin Psychol Rev 2013; 33(6): 763–71. 23796855 10.1016/j.cpr.2013.05.005

[3] Strauss C, Cavanagh K, Oliver A, Pettman D. Mindfulness-based interventions for people diagnosed with a current episode of an anxiety or depressive disorder: a meta-analysis of randomised controlled trials. PLoS One 2014; 9(4): e96110. 24763812 10.1371/journal.pone.0096110 PMC3999148

[4] Goyal M, Singh S, Sibinga E, Gould N, Rowland-Seymour A, Sharma R, Meditation programs for psychological stress and well-being. JAMA Intern Med 2014; 174(3): 357. 24395196 10.1001/jamainternmed.2013.13018 PMC4142584

[5] Jonsson U, Alaie I, Parling T, Arnberg F. Reporting of harms in randomized controlled trials of psychological interventions for mental and behavioral disorders: a review of current practice. Contemp Clin Trials 2014; 38(1): 1–8. 24607768 10.1016/j.cct.2014.02.005

[6] Schlosser M, Jones R, Demnitz-King H, Marchant N. Meditation experience is associated with lower levels of repetitive negative thinking: the key role of self-compassion. Curr Psychol [Epub ahead of print] 9 Jun 2020. Available from: 10.1007/s12144-020-00839-5.

[7] Britton WB, Lindahl JR, Cooper DJ, Canby NK, Palitsky R. Defining and measuring meditation-related adverse effects in mindfulness-based programs. Clin Psychol Sci 2021; 9(6): 1185–204. 10.1177/2167702621996340 PMC8845498 35174010

[8] Goldberg SB, Lam SU, Britton WB, Davidson RJ. Prevalence of meditation-related adverse effects in a population-based sample in the United States. Psychother Res [Epub ahead of print] 2 Jun 2021. Available from: 10.1080/10503307.2021.1933646. PMC8636531 34074221

[9] Farias M, Maraldi E, Wallenkampf KC, Lucchetti G. Adverse events in meditation practices and meditation-based therapies: a systematic review. Acta Psychiatr Scand 2020; 142(5): 374–93. 32820538 10.1111/acps.13225

[10] Duggan C, Parry G, McMurran M, Davidson K, Dennis J. The recording of adverse events from psychological treatments in clinical trials: evidence from a review of NIHR-funded trials. Trials 2014; 15: 335. 10.1186/1745-6215-15-335 PMC4152561 25158932

[11] World Health Organization. Conceptual Framework for the International Classification for Patient Safety. World Health Organization, 2010 (https://www.who.int/patientsafety/taxonomy/icps_full_report.pdf).

[12] Linden M. How to define, find and classify side effects in psychotherapy: from unwanted events to adverse treatment reactions. Clin Psychol Psychother 2012; 20(4): 286–96. 22253218 10.1002/cpp.1765

[13] Ehring T, Zetsche U, Weidacker K, Wahl K, Schönfeld S, Ehlers A. The Perseverative Thinking Questionnaire (PTQ): validation of a content-independent measure of repetitive negative thinking. J Behav Ther Exp Psychiatry 2011; 42(2): 225–32. 21315886 10.1016/j.jbtep.2010.12.003 PMC3042595

[14] Schlosser M, Sparby T, Vörös S, Jones R, Marchant N. Unpleasant meditation-related experiences in regular meditators: prevalence, predictors, and conceptual considerations. PLoS One 2019; 14(5): e0216643. 31071152 10.1371/journal.pone.0216643 PMC6508707

[15] Baer R, Smith G, Allen K. Assessment of mindfulness by self-report. Assessment 2004; 11(3): 191–206. 15358875 10.1177/1073191104268029

[16] Costa P, McCrae R. The five-factor model, five-factor theory, and interpersonal psychology. In Handbook of Interpersonal Psychology: Theory, Research, Assessment, and Therapeutic Interventions (eds LM Horowitz, S Strack): 91–104. Wiley, 2010.

[17] Crescentini C, Capurso V. Mindfulness meditation and explicit and implicit indicators of personality and self-concept changes. Front Psychol 2015; 6: 44. 10.3389/fpsyg.2015.00044 PMC4310269 25688222

[18] Smith T, Glazer K, Ruiz J, Gallo L. Hostility, anger, aggressiveness, and coronary heart disease: an interpersonal perspective on personality, emotion, and health. J Pers 2004; 72(6): 1217–70. 15509282 10.1111/j.1467-6494.2004.00296.x

[19] Dahl C, Lutz A, Davidson R. Reconstructing and deconstructing the self: cognitive mechanisms in meditation practice. Trends Cogn Sci 2015; 19(9): 515–23. 26231761 10.1016/j.tics.2015.07.001 PMC4595910

[20] Lindahl J, Cooper D, Fisher N, Kirmayer L, Britton W. Progress or pathology? Differential diagnosis and intervention criteria for meditation-related challenges: perspectives from Buddhist meditation teachers and practitioners. Front Psychol 2020; 11: 1905. 10.3389/fpsyg.2020.01905 PMC7403193 32849115

[21] Brown K, Ryan R. The benefits of being present: mindfulness and its role in psychological well-being. J Pers Soc Psychol 2003; 84(4): 822–48. 12703651 10.1037/0022-3514.84.4.822

[22] Black D, Sussman S, Johnson C, Milam J. Psychometric assessment of the Mindful Attention Awareness Scale (MAAS) among Chinese adolescents. Assessment 2011; 19(1): 42–52. 21816857 10.1177/1073191111415365 PMC3705937

[23] John OP, Srivastava S. The Big-Five trait taxonomy: history, measurement, and theoretical perspectives. In Handbook of Personality: Theory and Research (eds LA Pervin, OP John): 102–38. Guilford Press, 1999.

[24] Waddell J, Nissen L, Hale A, Kyle G. Using the Big Five inventory to evaluate the personality traits of Australian pharmacists. Int J Pharm Pract 2019; 28(3): 275–81. 31828855 10.1111/ijpp.12597

[25] Anderson T, Suresh M, Farb NAS. Meditation benefits and drawbacks: empirical codebook and implications for teaching. J Cogn Enhanc 2019; 3(2): 207–20.

[26] Cebolla A, Demarzo M, Martins P, Soler J, Garcia-Campayo J. Unwanted effects: is there a negative side of meditation? A multicentre survey. PLoS One 2017; 12(9): e0183137. 28873417 10.1371/journal.pone.0183137 PMC5584749

[27] Lomas T, Cartwright T, Edginton T, Ridge D. A qualitative analysis of experiential challenges associated with meditation practice. Mindfulness 2014; 6(4): 848–60.

[28] Vieten C, Wahbeh H, Cahn B, MacLean K, Estrada M, Mills P, Future directions in meditation research: recommendations for expanding the field of contemplative science. PLoS One 2018; 13(11): e0205740. 30403693 10.1371/journal.pone.0205740 PMC6221271

[29] Morone N, Moore C, Greco C. Characteristics of adults who used mindfulness meditation: United States, 2012. J Altern Complement Med 2017; 23(7): 545–50. 28084788 10.1089/acm.2016.0099

[30] McEvoy PM, Brans S. Common versus unique variance across measures of worry and rumination: predictive utility and mediational models for anxiety and depression. Cognit Ther Res 2013; 37(1): 183–96.

[31] Barrick M, Mount M, Judge T. Personality and performance at the beginning of the new millennium: what do we know and where do we go next? Int J Sel Assess 2001; 9(1&2): 9–30.

[32] Sauer-Zavala S, Wilner J, Barlow D. Addressing neuroticism in psychological treatment. Personal Disord 2017; 8(3): 191–8. 29120218 10.1037/per0000224

[33] Linehan M. DBT Skills Training Manual 2nd ed. Guilford Publications, 2014.

[34] Segal Z, Williams J, Teasdale J. Mindfulness Based Cognitive Therapy for Depression. Guilford Press, 2002.

[35] Böge K, Karadza A, Fuchs L, Ehlen F, Ta T, Thomas N, Mindfulness-based interventions for in-patients with schizophrenia spectrum disorders—a qualitative approach. Front Psychiatry 2020; 11: 600. 32676042 10.3389/fpsyt.2020.00600 PMC7333646

